# Understanding for whom, why and in what circumstances payment for performance works in low and middle income countries: protocol for a realist review

**DOI:** 10.1136/bmjgh-2017-000695

**Published:** 2018-06-27

**Authors:** Josephine Borghi, Neha S Singh, Garrett Brown, Laura Anselmi, Soren Kristensen

**Affiliations:** 1 Department of Global Health and Development, London School of Hygiene and Tropical Medicine, London, UK; 2 School of Politics and International Studies, University of Leeds, Leeds, UK; 3 Manchester Centre for Health Economics, University of Manchester, Manchester, UK; 4 Faculty of Medicine, Institute of Global Health Innovation, Centre for Health Policy, Imperial College, London, UK

**Keywords:** health systems, health systems evaluation, review

## Abstract

**Background:**

Many low and middle income countries (LMIC) are implementing payment for performance (P4P) schemes to strengthen health systems and make progress towards universal health coverage. A number of systematic reviews have considered P4P effectiveness but did not explore how P4P works in different settings to improve outcomes or shed light on pathways or mechanisms of programme effect. This research will undertake a realist review to investigate how, why and in what circumstances P4P leads to intended and unintended outcomes in LMIC.

**Methods:**

Our search was guided by an initial programme theory of mechanisms and involved a systematic search of Medline, Embase, Popline, Business Source Premier, Emerald Insight and EconLit databases for studies on P4P and health in LMIC. Inclusion and exclusion criteria identify literature that is relevant to the initial programme theory and the research questions underpinning the review. Retained evidence will be used to test, revise or refine the programme theory and identify knowledge gaps. The evidence will be interrogated by examining the relationship between context, mechanisms and intended and unintended outcomes to establish what works for who, in which contexts and why.

**Discussion:**

By synthesising current knowledge on how P4P affects health systems to produce outcomes in different contexts and to what extent the programme design affects this, we will inform more effective P4P programmes to strengthen health systems and achieve sustainable service delivery and health impacts.

**Summary box**

- Previous reviews of payment for performance (P4P) have focused on programme effectiveness, finding mixed effects on incentivised outcomes. These reviews did not systematically examine how P4P works in different contexts and its potential unintended effects.

- This article presents the protocol for a realist review to shed light on for who, how and in what circumstances P4P works in low and middle income countries (LMIC). This review will increase our understanding of how P4P affects health systems and how health systems can be strengthened to deliver better care and key attributes of successful P4P programmes in different contexts.
- The outcome of the review will be a refined middle range programme theory reflecting actor response to P4P (mechanisms) and contextual factors shaping this, that can be generalised across LMIC settings.

- The review will inform the design of more effective P4P programmes to strengthen health systems for specific contexts and minimise unintended negative effects.

## Background

There has been growing international commitment in recent years to the goal of achieving universal health coverage (UHC) or access to affordable and effective care. UHC relies on strong health systems^1^ and on the effective and equitable allocation and use of available resources. However, health systems in many settings struggle to deliver quality care due, for example, to inadequate infrastructure; lack of motivated staff; a lack of drugs and medical supplies and limited adherence to clinical care guidelines.^2^

Payment for performance (P4P) has been proposed as a strategy to strengthen health systems, to improve service delivery and population health. P4P consists of financial incentives to healthcare providers and/or their managers based on service delivery performance^3 4^ and assumes individuals exert more effort in response to incentives, which will result in improved quality of care. P4P has been widely applied in the USA and the UK.^5–7^ In these countries, payers most often introduce P4P by targeting the organisational level rather than the individual provider level, with a view to improving quality of care. Literature from these settings suggests that while P4P may improve processes of care, very few studies have demonstrated improvements in health outcomes.^8^ Calls for a better understanding of P4P mechanisms have also been voiced 9 10 and some authors are now suggesting a reconsideration of the role of P4P as a means of improving outcomes in healthcare.^11^ Over 30 low and middle income countries (LMIC) are currently implementing P4P schemes in the health sector with financial support from international donors.^12^ In LMIC, P4P can involve additional resources to improve service delivery, investments in information systems, more frequent supervision of healthcare providers and greater financial decentralisation.^13^ Hence, in these settings, P4P represents a complex package of interventions, aimed at strengthening health systems to deliver better care.^14^ The focus of incentives in these settings is on the quantity of services delivered and quality of care.

Most early evaluations of P4P schemes in LMIC considered programme effectiveness in terms of improving patient and population health outcomes. A systematic review of the effect of P4P programmes in LMIC published in 2012 reported that the evidence base was too limited to draw overall conclusions and that more attention was needed to understand how incentive design impacts on programme effectiveness.^3^ This review is currently being updated. A more recent review of the effect of P4P on quality found evidence of improved process quality for antenatal care, but limited or mixed evidence for other quality indicators.^15^ A third review examined the effects of P4P on HIV indicators, identifying only four studies with positive effects on coverage of HIV testing for couples and pregnant women and coverage of antiretrovirals in pregnant women, infants and adults.^16^

In more recent years, researchers have recognised the importance of also understanding change mechanisms^17^ and documenting the effects of P4P on the health system. As a result, there is growing evidence of the effects of P4P on health workers,^18 18–24^ accountability mechanisms including supervision systems and community engagement in service delivery,^25 26^ information systems and use of data,^27^ drug availability,^28^ patient satisfaction and provider-patient interactions.^5^ None of this evidence is captured within the systematic reviews referenced above. A more recent review did summarise much of this evidence;^13^ however, the review was focused on extracting policy recommendations, but did not explore how P4P works in different settings to bring about improvements in outcomes or shed light on the pathways or mechanisms of programme effect.

In light of emerging evidence of the health system effects of P4P, a further review of the literature is needed to investigate how, why and in what circumstances P4P leads to intended and unintended outcomes in LMIC and how the design of P4P incentives affects the way P4P programmes work and their outcomes. Such a review will increase our understanding of how P4P affects health systems and how health systems can be strengthened to deliver better care and key attributes of successful P4P programmes in different contexts.

The choice of synthesis methodology should be driven by the research question^29–32^ and we contend that a realist approach is best suited for such a review. A systematic review is a robust methodology for assessing whether interventions are effective, but is not suited to unpacking or explaining effects or to answering ‘how and why’ questions.^33 34^ Indeed, the existing systematic reviews on P4P only included experimental or quasi-experimental studies addressing the question of how effective the programme was and did not document process changes or contextual factors which allowed the interventions to produce outcomes. In contrast to systematic reviews, realist reviews are able to include and synthesise a much broader set of evidence, including qualitative methods which address ‘how and why’ questions. A realist approach assumes that complex interventions do not operate in a silo; rather they operate within social systems and it is ‘the mechanism’ (the response and behaviour of agents interacting within the social system) that determines outcomes within a given context.^34 35^ The context can be social, cultural, historical or institutional and is what facilitates or limits the action of agents.^36^ The realist approach is guided by an initial programme theory of how the programme is expected to lead to given outcomes, why and in what context (referred to as a context-mechanism-outcome configuration—CMO). The realist approach tries to then empirically test the hypothesised ‘mechanisms’ or the way actors are expected to respond to P4P programmes and the changes their response brings about that leads to intended and unintended outcomes and to assess how this mechanism varies according to the context of programme implementation. The review findings are then used to determine which CMO configuration(s) offer the most robust and plausible explanation of observed outcomes. This resulting CMO configuration is then compared with the initial programme theory, which is modified in light of these findings, resulting in a ‘middle range’ programme theory (or a robust understanding of the sets of mechanisms and how they unfold in different contexts), which can be generalised across LMIC.^37^ In this way, the realist approach aims to discern what works for whom, in what context and how and why it works to produce both intended and unintended outcomes.^37^

## Methods

### Aim and objectives

The overall aim of this review is to help researchers and policy makers understand how and why P4P programmes implemented in low and middle income countries result in intended or unintended outcomes, how the context within which they are implemented affects this and which incentive designs are most effective. In so doing, the review will produce a refined middle range programme theory for P4P reflecting the way the programme works to deliver specific outcomes in different contexts that can be generalised across LMIC. The review aims to address the following specific questions:

1. How do actors respond to P4P programmes and what changes does their response bring about that leads to intended and unintended outcomes (what is the ‘mechanism’ through which the programme affects outcomes)?
2. What contextual and programme design factors determine whether the identified ‘mechanisms’ produce these outcomes?

### Study design

We will carry out a realist review as proposed by Pawson and colleagues.^37^ The study will be conducted in six steps, namely: (1) developing initial programme theory; (2) searching for evidence; (3) selecting and appraising documents; (4) extracting data; (5) synthesising evidence; (6) presenting and disseminating middle range programme theory, as outlined in table 1 and detailed below. At the time of writing, some of the first steps had already been initiated or completed. As a result, we use the past tense to describe steps that have been completed and the future tense to describe steps that have yet to start.

**Table 1:** Methodological steps to complete the realist review (adapted from Molnar *et al*^60^)

	Steps	Task(s)
1	Clarifying the initial programme theory	Search for initial theories and then consult with experts
2	Search strategy	Search electronic databases using keywords and Medical Subject Heading (MeSH) terms
3	Select and appraise documents	Use inclusion and exclusion criteria to screen for relevant abstracts, articles and reports Retrieve full-text of articles and reports
4	Extract data	Use standardised tool to extract relevant data Search reference lists by hand for additional potentially relevant articles and reports
5	Analysis and synthesis process	Analyse data for content and outcome patterns and synthesise mechanisms NB: Realist reviews follow an iterative search process, so revise Step 2 (ie, search strategy) if relevant
6	Present and disseminate revised programme theory	Present and refine revised theoretical findings with relevant stakeholders and experts

#### Step 1—Developing an initial programme theory

This step aims to develop an initial programme theory for P4P: (1) highlighting the anticipated response of actors to the P4P programme and how this response translates into (intended and unintended) changes in outcomes and (2) identifying the contextual factors ((institutional, organisational, socioeconomic, cultural) influencing the actor response to the programme (the ‘mechanism’). This step also aims to present this theory visually within a diagram. To develop an initial programme theory for P4P, we drew on five sources of information: (1) motivation theories and theories of demand; (2) existing published theories of change related to P4P that were known to the authors; (3) theories of change developed during stakeholder workshops convened by the research team; (4) the research team’s (JB, GB, LA) own research knowledge and experience related to P4P; (5) existing reviews of P4P.

Existing bodies of theory were used to support the general development of the initial programme theory in relation to provider response to incentives (motivation theories of how workers respond to incentives^38–40^) and patient response to changes in service provision (the Grossman theory of demand^41^). To tailor the initial programme theory more specifically to P4P, two published theories of change were then identified and appraised: that presented in the World Bank’s P4P impact evaluation toolkit^12^ and a theory of change used within an evaluation study of P4P in Tanzania, in which two of the researchers were involved (JB, LA).^42^ Two theories of change diagrams were also reviewed that resulted from stakeholder workshops: the first developed by researchers studying P4P in LMIC, who attended a Resilient and Responsive Health Systems research consortium workshop in Dar es Salaam, Tanzania in November 2015 (including JB and LA) and the second developed by Mexican and UK researchers (including JB and LA) studying financial incentives in health, during a Newton funded Researcher Links UK-Mexico workshop in April 2015. To derive our initial programme theory for this review and construct an associated diagram, two members of the review team (JB and NSS) appraised each diagram, giving priority to common pathways and considered these in relation to existing knowledge within the team and evidence from published reviews.

**Figure 1:**
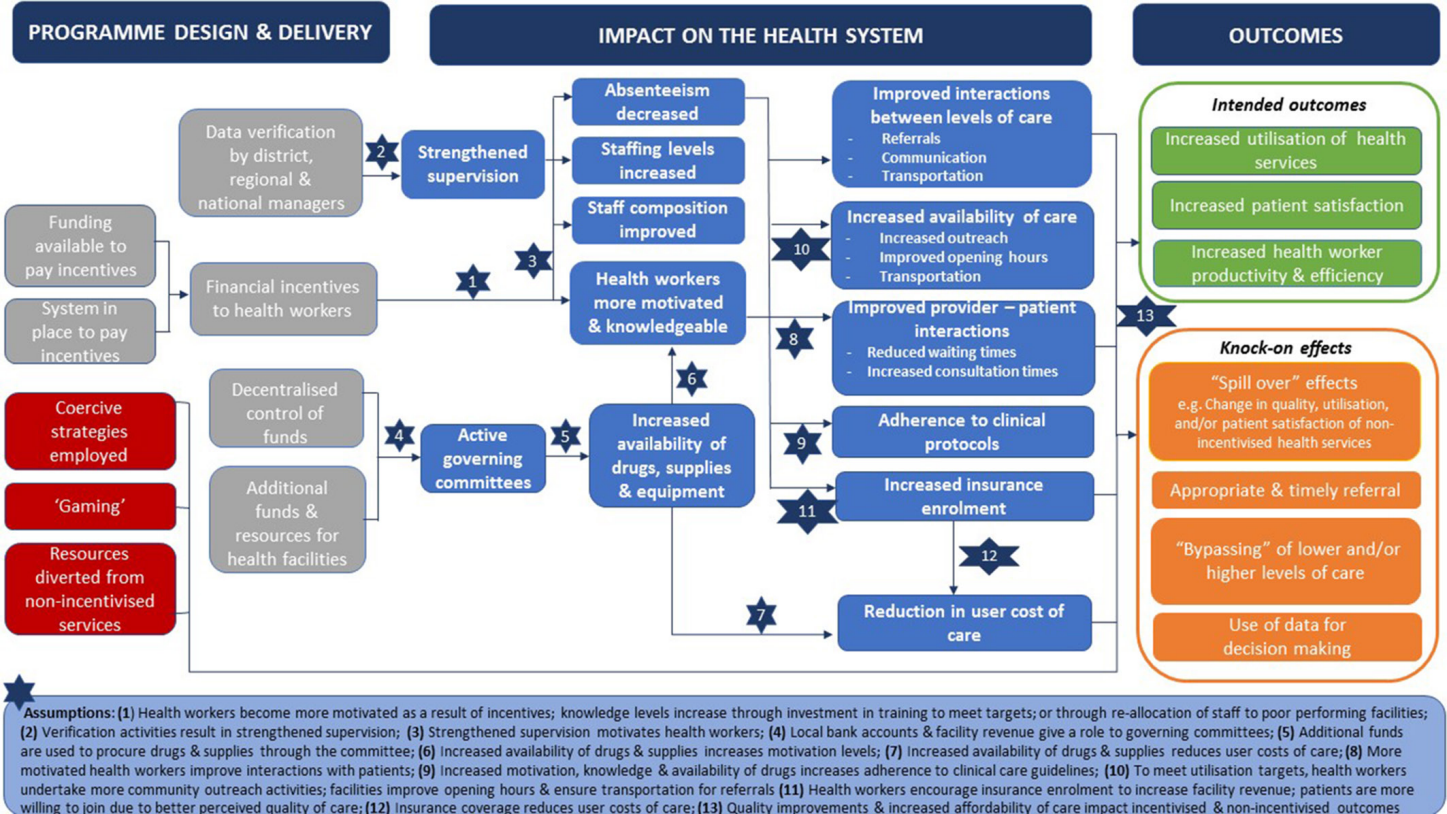
Initial programme theory mapping the mechanisms through which P4P affects the health system and results in outcomes.

The resulting diagram, figure 1, provides a visual representation of our initial programme theory. In summary, the direct effect of individual financial incentives is to make health workers more motivated to adhere to the incentivised dimensions of the service (obtain training to provide this care, increasing knowledge) and to adopt strategies to attract patients to facilities for incentivised services (for quantity targets) to maximise incentive payments.^43–45^ Actions taken to comply with incentivised indicators may include increased adherence to the clinical care guidelines, making services more affordable (reducing informal charges, boosting insurance enrolment^46^), making services more available (longer opening hours; increased outreach care^47^) and becoming more responsive to patient needs (improved client-provider interactions^15 46^), resulting in greater patient satisfaction, greater community engagement in service delivery through facility governing committees^48^). Other components of P4P programmes are also expected to result in change.^49^ The need for managers to verify performance data may increase interactions between providers and managers, and when the latter are incentivised, strengthen relations between levels of the system, including referrals, and result in more frequent and focused supportive supervision.^26^ Greater commitment of managers to service delivery may increase the use of data for decision making and the prioritisation of resources to maximise performance on incentivised indicators (eg, through increased staffing levels and improved staff composition^50^ and facilitating provider access to drugs and supplies). Part of the incentive is provided to the facility for investment in drugs, medical supplies or equipment, the availability of which would conceivably increase in response to the programme^28^ and in turn would enhance worker motivation and ability to deliver better and more affordable care (by reducing user charges), increasing patient satisfaction and service utilisation. However, P4P can also result in unintended consequences such as misreporting performance (gaming),^51 52^ the use of coercive strategies to boost demand,^47^ a reluctance to refer or treat patients that could negatively affect their performance score, a displacement of effort away from un-incentivised services^53^ and positive spillover effects.^46^

As contextual factors were not identified within the theory of change diagrams we reviewed and appraised, these were not included in our diagram. However, we identify a number of potentially relevant contextual factors within our initial programme theory, notably: other policies especially those operating on the demand side might impact on P4P effectiveness; the level of health system performance and capacity at the time of introduction of P4P and access to other funding streams^54^; the organisational culture within which P4P is introduced, management competencies; the wider context of health worker pay^54^ and the population culture and attitudes towards formal medical care.^55^ We will also explore whether effects differ in fragile or postconflict settings, compared with other settings,^3^ and according to how P4P was introduced (embedded in existing government purchasing systems or as a stand alone programme). We will look for details of context within the reviewed papers (in relation to characteristics of populations covered by P4P; providers implementing P4P and the broader (institutional, economic, geographic, cultural) context within which it is implemented.

We will look for evidence of how contextual factors affect implementation and programme mechanisms and through comparison across studies, we will examine whether mechanisms and outcomes are associated with any dimensions of the context within which they are implemented. Some aspect of programme design have also been identified as potentially relevant to understanding mechanisms and outcomes (specifically the level of incentive relative to other funding, and who and what is and is not incentivised).^3 55^

The initial programme theory was presented to a policy and academic audience at the Fourth Global Forum for Human Resources in Health in November 2017 for external validation. The programme theory and diagram will be revisited throughout the evidence review process and revised to reflect emerging findings.

#### Step 2—Searching for evidence

In a second step, we conducted a systematic search for primary studies that are relevant to the programme theory set out in Step 1, with the aim of identifying evidence to test and refine the programme theory. We searched Medline, Embase, Popline, Business Source Premier, Emerlad Insight and EconLit databases. A search strategy was developed and carried out in collaboration with an experienced university librarian. The search included appropriate indexing terms (ie, MeSH terms and keywords) on P4P; on mechanisms relating to programme theory and geographic focus (eg, low and middle income countries as defined by the World Bank^56^). The search strategy was first developed in Medline and then adapted to the other databases (online supplementary appendix 1). The results of the initial search were reviewed to examine whether any known references were excluded. The search strategy was subsequently modified to be more inclusive and ensure the retention of all known articles of relevance by searching for P4P MeSH terms and keywords and restricting to low and middle-income countries as defined by the World Bank.^56^ The search period covered 1 January 1995, when empirical literature first started to emerge on P4P in LMIC, to 21 November 2017.

10.1136/bmjgh-2017-000695.supp1 Supplementary file 1

After screening papers identified in the systematic search, we will also search for international unpublished and grey literature (eg, websites of key stakeholders including: World Bank, the World Health Organization, Cordaid, Norad, the Department for International Development, USAID and PEPFAR) as well as Google Scholar and Web of Science to identify academic working papers and evaluation reports or policy documents published by LMIC governments, international organisations, non-governmental organisations and consultancy firms. The research team’s contacts with networks of researchers, decision-makers and other stakeholders with knowledge of P4P in LMIC will also facilitate the identification and collection of such documents.

Reference lists of all identified studies and documents will be screened to identify potential additional literature that were not captured within the original review. The literature review search will end at the point of saturation, that is, when the research yields no further new sources of information. All search results from electronic databases and other sources will be imported into Endnote reference management software and duplicates removed.

#### Step 3—Selecting and appraising documents

Inclusion and exclusion criteria were developed to identify literature that is relevant to the initial programme theory (figure 1) and the research questions underpinning the review^57^—see table 2. Retained evidence will be used to test, revise or refine elements within the initial programme theory. The review will also identify knowledge gaps where evidence is limited in relation to the initial programme theory. Where there is conflicting evidence on a given component, we will explore contextual and scheme design differences that may account for variation in findings. Studies with any evaluation design were allowed as per realist review guidelines. The relevance of the retrieved articles/publications was assessed according to the inclusion and exclusion criteria outlined below. Next, two reviewers (NSS and JB) independently screened all titles and abstracts for suitability for inclusion. Disagreements were noted and discussed to reach agreement based on consistent criteria.

**Table 2:** Inclusion and exclusion criteria

Inclusion criteria	Exclusion criteria
The exposure (intervention) is a P4P intervention. These schemes had to target healthcare providers and/or managers (supply-side), incentives had to be financial and cash disbursement had to be varied accordingly to performance, defined as the achievement of quantitative indicators for selected healthcare services, quality-related indicators or both quantity-related and quality-related indicators. When performance is linked to quantitative outputs, it has to be related to ‘selected healthcare services’ as such criterion allows one to discriminate between P4P and fee for service mechanisms.	Schemes targeting beneficiaries of health services (ie, demand-side incentives); Supply-side P4P schemes outside of the health sector; P4P schemes that did not specifically target selective health services and, therefore, were not distinguishable from fee for service schemes
The study should isolate (or attempt to isolate) the effects of P4P programmes from that of broader policy reforms, which often encompass the P4P reform.	Studies which do not attempt to isolate the effects of P4P from broader reforms.
The evaluation of pilot projects will be included in the study.	Studies of the ‘potential’ implementation of P4P strategies not yet in place.
The study outcome has to be either a quantitative or qualitative measure (or both) of the impact of the P4P initiative on one or more health system functions described in the initial programme theory (figure 1) or other relevant mechanisms or contextual factors affecting outcomes.	Studies that only examine impact on ultimate service utilisation and health outcomes and do not examine mechanisms and/or context.
The study should report on primary data sources. Where the study refers to different sources of evidence for primary data (eg, in the case of systematic reviews), the primary source of information will be retrieved and explored.	Studies that do not report primary data sources (eg, commentaries or reviews), though these will be screened for relevant additional references.
The intervention had to be implemented in an LMIC, as defined by the World Bank.^56^	Studies in high-income settings.
Studies in English, French, Portuguese or Spanish.	Studies in languages other than English, French, Portuguese or Spanish

FFS, fee-for-service; LMIC, low and middle income countries; P4P, payment for performance.

#### Step 4—Extracting data

Data will be extracted into a Microsoft Excel spreadsheet. The spreadsheet captures information on general study characteristics such as the author name, year, study setting, P4P implementing organisation, and study type. In addition, data will be extracted according to a set of domains identified by NSS and JB based on the content of the initial programme theory, documenting mechanisms (the programme effects on different health system elements and the links between these effects), context (who is this working for or not working for; why is this working or not working; in what contexts is it working/not working), outcomes (the final outcomes of the programme) and programme design elements (level of incentive, what is incentivised, who gets the incentive). To test the suitability of these domains they will be pretested on two purposefully selected articles by NSS and JB. The domains will be revised iteratively as the review progresses and new mechanisms and effects emerge from the evidence that were not within the original programme theory and as more focused and specific research questions arise.

We will also appraise the quality of methodology and describe the methodology using The Mixed-Method Appraisal Tool, which is suited to the assessment of quantitative, qualitative and mixed-methods approaches.^58^

The review will compare and contrast evidence, looking for consistent CMO configurations emerging from the evidence to support, refine or modify the initial programme theory.

#### Step 5—Analysis and synthesis of data

Each study will be read by two authors (JB and NSS) and discussed to assess whether emerging findings support, refute or reinterpret the preliminary programme theory (figure 1). To this end, evidence will be interrogated by examining the relationship between context, mechanisms and outcomes, both intended and unintended, to establish what works for who, in which contexts and why. The analysis and synthesis will involve looking for recurrent relationships between context, mechanisms and outcomes in the documents. We will examine how similar mechanisms act in different contexts to generate outcomes. The findings emerging from the review will be questioned and contradictory examples sought in the data. An appraisal of the strengths/weaknesses of the research methods used by papers to be integrated into the synthesis will be undertaken. The findings will be used to refine, adjust or modify theory, resulting in a middle-range theory^37^ of the links between contextual factors, mechanisms and outcomes of P4P interventions.

#### Step 6—Generating a revised programme theory

The revised programme theory will be described in text and using a diagram and the review will be reported according to the standards outlined by the realist and meta-narrative evidence synthesis (RAMESES) group.^59^ Pawson *et al*^37^ argue that stakeholders should be involved in confirming emerging findings and in dissemination activities. Emerging findings and CMO configurations will be presented and debated during a conference session on P4P theories of change in 2018. The final programme theory will be that emerging from these stakeholder discussions.

## Discussion

This study involves a realist approach to synthesising evidence to generate an improved understanding of how and why P4P programmes result in intended or unintended outcomes, within which contexts and which incentive designs are most effective. In so doing, the review will ultimately produce a refined middle range programme theory for P4P reflecting actor response to P4P (mechanisms) and contextual factors shaping these, that can be generalised across LMIC. The use of a realist approach will allow the review to describe and explain how and why P4P initiatives work (or fail to work) in different contexts by exploring the underlying programme theories and the interactions between contextual factors, mechanisms of change and outcomes.

Synthesising current knowledge of how P4P affects health systems to produce outcomes in different contexts and to what extent the incentive design affects this will inform more effective P4P programmes to strengthen health systems and achieve sustainable service delivery and health impact and minimise unintended effects. This review will also shed light on how context shapes the design of performance-based financing programmes and their subsequent implementation, which will be useful in determining where P4P can be most effectively implemented.
