# Supplementary material for: Understanding for whom, why and in what circumstances payment for performance works in low and middle income countries: protocol for a realist review
Source: BMJ Glob Health. 2018 Jun 27;3(3):e000695. doi: 10.1136/bmjgh-2017-000695 (PMC6035508; doi:10.1136/bmjgh-2017-000695)
Supplement: Supplementary file 1 [file bmjgh-2017-000695supp001.pdf]

Supplementary file: Search terms and syntax

Payment for performance (P4P)

1. “Physician incentive plans” [Mesh] OR “Employee incentive plans’[Mesh]OR “Reimbursement, incentive”[Mesh] OR “Fee-for-service plans”[Mesh] OR “Contract services”[Mesh]
2. (conditional adj3 “pay* or transfer?”)[tiab] OR (pay* adj3 performance)[tiab] OR p4p[tiab] OR pfp[tiab] OR (pay* adj3 quality)[tiab] OR “fee? for service?”[tiab] OR ((incentive? or compensatory or reimbursement) adj plan?)[tiab] OR “contract service” [tiab] OR “result? based financing”[tiab] OR “performance based financing”[tiab] OR ((result? or performance or output or out put) adj2 “finance* or fund* or pay* or disburs* or fee? or incentive? or initiative? or contract? or aid”)[tiab] OR “payment incentive”[tiab] OR “monetary incentive*”[tiab] OR “economic incentive*”[tiab] OR “financial incentive*”[tiab] OR “reimbursement incentive*”[tiab] OR ((economic or financial) adj (reward? or bonus*))[tiab] OR “target* pay*”[tiab] or “provider* performance”[tiab] or “provider* incentive*”[tiab] OR “pay for value”[tiab] OR “pay* for quality”[tiab] OR “performance-based pay*”[tiab] OR “performance-based reimbursement”)[tiab] OR ((doctor? or "healthcare provider?" or "health care provider?" or nurse? or nursing or physician? or (practitioner?) adj4 (bonus* or incentive? or financial or monetary* or payment? or pay? or plan? or reward? or salar*))[tiab] OR ("pay for quality improvement?" or P4QI or "fee-for service?")[tiab] OR ("performance based" or value-based)[tiab] OR ((financial or incentive? or monetary* or physician? or provider? or practitioner? or salar*) adj2 (bonus* or reward?))[tiab] OR “performance-based contracting”[tiab] OR “output-based payment”[tiab] OR “incentive program”[tiab] OR “quality-based purchasing”[tiab] OR “quality incentive”[tiab] OR “quality-payment*”[tiab] OR “quality-based payment*”[tiab] OR (quality-based[tiab] AND payments[tiab]) OR (Employee Incentive Plans[Mesh] AND (finance* or pay* or incentive? or initiative? or bonus*) [tiab]) OR (Physician Incentive Plans[Mesh] AND (finance* or pay* or incentive? or initiative? or bonus*)[tiab]) or (Reimbursement, Incentive[Mesh] AND (finance* or pay* or incentive? or initiative? or bonus*)[tiab])
3. #1 OR #2

Medicines

1. (drug[ti] or drugs[ti] or pharmaceutical[ti] or pharmaceuticals[ti] or medicines[ti] or medicine[ti])
2. (drug[tiab] or drugs[tiab] or pharmaceutical[tiab] or pharmaceuticals[tiab] or medicines[tiab] or medicine[tiab])
3. (pharmaceutical[ti] or pharmaceuticals[ti] or medicines[ti])
4. (pharmaceutical[tiab] or pharmaceuticals[tiab] or medicines[tiab])

A. **<u>Insurance and Financing</u>**

5. ("Drug Costs/legislation and jurisprudence"[Mesh] OR " Economics, Pharmaceutical/legislation and jurisprudence"[Mesh] OR "Fees, Pharmaceutical/legislation and jurisprudence "[Mesh])
6. "Rate Setting and Review/legislation and jurisprudence"[Mesh] AND (#2)
7. (reference[ti] OR referencing[ti] AND (price[ti] OR prices[ti] OR pricing[ti])) OR (maximum[ti] AND (price[ti] OR prices[ti] OR pricing[ti])) OR (internal[ti] OR external[ti] AND (price[ti] OR prices[ti] OR pricing[ti]))
8. ("insurance, health, reimbursement"[Mesh] OR "Reimbursement Mechanisms"[Mesh] OR "Cost Sharing"[Mesh]) AND (#4)
9. (copay[ti] OR copays[ti] OR copayment[ti] OR "co pay"[ti] OR "co payment"[ti] OR "co payments"[ti] OR "fees"[ti]) AND (#4)

B. **<u>Prescribing and Use</u>**

10. "Drug Prescriptions/legislation and jurisprudence"[Mesh]
11. "Practice Guidelines as Topic/standards"[Majr] AND (#3)
12. "Drugs, Generic "[MAJR] and (substitute[tiab] or substitution[tiab] or substitutions[tiab])
13. "Drug Utilization/legislation and jurisprudence"[Mesh]
14. "rational use"[ti] and (pharmaceutical[tiab] or pharmaceuticals[tiab] or medicines[tiab])

C. **<u>Medicines access</u>**

15. "Health Services Accessibility"[MAJR] AND (#3)

D. Medicines supply management

16. "prescription Drugs/supply and distribution"[Mesh] OR "Nonprescription Drugs/supply and distribution"[Mesh] OR ("Drug Storage/methods"[Majr] OR "Drug Storage/standards"[Majr])

Medicines (All Categories)

17. (#5) OR (#6) OR (#7) OR (#8) OR (#9) OR (#10) OR (#11) OR (#12) OR (#13) OR (#14) OR (#15) OR (#16)

18. "Drug Abuse"[tiab] OR "Drug Possession"[tiab] OR "narcotic abuse"[tiab] OR "narcotic use"[tiab] OR narcotics[tiab] OR "substance abuse"[tiab] OR poison[tiab] OR poisoning[tiab] OR venom[tiab] OR "substance use"[tiab] OR cocaine[tiab] OR heroin[tiab] OR marijauna[tiab] OR pot[tiab] OR "salvia divinorum"[tiab]

19. (#17) NOT (#18)

Health Financing

A. Payment mechanisms (not including P4P)

1. "Prepaid Health Plans"[Mesh] OR "Group Practice, Prepaid"[Mesh] OR "Capitation Fee"[Mesh] OR "Salaries and Fringe Benefits"[Mesh] OR "Financing, Organized"[Mesh] OR "Fees and Charges"[Mesh] OR "Fees, Medical"[Mesh]

B. Health insurance

2. "Insurance,Health" [Mesh] OR "Insurance Pools" [Mesh] OR "Insurance Coverage" [Mesh]

C. Resource Allocation

3. "Resource Allocation" [Mesh] OR "Health Care Rationing" [Mesh] Or "cost-benefit analysis"[Mesh]
4. “Pooling”[tiab] OR “Purchas*”[tiab] OR “budget*”[tiab] OR “account*”[tiab] OR “cost” [tiab] OR “affordable care” [tiab] OR “affordability” [tiab] OR “cost of care” [tiab] OR “decentrali?at*”[tiab] OR “exemption*”[tiab] OR “waiver*”[tiab] OR “out of pocket” [tiab] OR “out-of-pocket” [tiab] OR “user charge*”[tiab] OR “resource level*”[tiab] OR “funding level*”[tiab] OR “accounting”[tiab]

Health Financing (All Categories)

5. #1 OR #2 OR #3 OR #4

Human Resources for Health (HRH)

1. "health manpower" [MeSH]

2. “health personnel" [tiab] OR "health care personnel" [tiab] OR "healthcare personnel" [tiab] OR "medical personnel" [tiab] OR "health professional" [tiab] OR "health care professional" [tiab] OR "healthcare professional" [tiab] OR "medical professional" [tiab] OR "health worker" [tiab] OR "health care worker" [tiab] OR "healthcare worker" [tiab] OR "medical worker" [tiab] OR "health workforce" [tiab] OR "health care workforce" [tiab] OR "healthcare workforce" [tiab] OR "medical workforce" [tiab] or “human resource” [tiab] OR “doctor”[tiab] OR “medical doctor”[tiab] OR “nurse”[tiab] OR “midwi*”[tiab]

3. (#2) NOT (#1)

4. (#1) OR (#3)
5. “team*”[tiab] OR “tension*”[tiab] OR “conflict”[tiab] OR “autonomy”[tiab]
6. (#4) AND (#5)

A. <u>Motivation, knowledge, satisfaction, retention and distribution</u>

7. "Health Services Needs and Demand"[Mesh] OR "Health Care Rationing"[Majr] OR "Resource Allocation"[Majr] OR "Personnel Management"[Majr] OR "Personnel Administration, Hospital"[Majr] OR "Health Resources"[Majr] OR "Job Satisfaction"[Mesh] OR "Burnout, Professional" [Mesh] OR "Personnel Turnover" [Mesh] OR Personnel Staffing and Scheduling[Mesh] OR Career Mobility[Mesh] OR "Personnel Selection"[Mesh]

8. supply[tiab] OR shortage[tiab] OR capacity[tiab] OR employment[tiab] OR distribution[tiab] OR maldistribution[tiab] OR maldistributions[tiab] OR recruit[tiab] OR recruite[tiab] OR recruitement[tiab] OR recruiting[tiab] OR allocation[tiab] OR reallocation[tiab] OR mobility[tiab] OR practice[tiab] OR rural[tiab] OR remote[tiab] OR underserved[tiab] OR (imbalance[tiab] OR retention[tiab] OR "retention strategy"[tiab] OR "financial incentive"[tiab] OR "monetary incentive"[tiab] OR allowances[tiab] OR benefits[tiab] OR "compulsory service"[tiab] OR "bonding scheme"[tiab] OR "vacancy rates"[tiab] OR motivation[tiab] OR (“Health Manpower”[Mesh] AND (recruitment or reten* or motivation or turnover or engagement or productivity or absenteeism or satisfaction or efficiency or performance or guideline* or behavi?r or quality)[tiab]) OR (“Medical Staff”[Mesh] AND (recruitment or reten* or knowledge or motivation or turnover or engagement or productivity or absenteeism or satisfaction or efficiency or performance or guideline* or behavi?r or quality)[tiab]) OR (“Nursing Staff”[Mesh] AND (recruitment or reten* or motivation or turnover or engagement or productivity or absenteeism or satisfaction or efficiency or performance or guideline* or behavi?r or quality)[tiab]) OR ((workforce or resources or staff or professional*) adj5 (recruitment or retention or motivation or turnover or engagement or productivity or absenteeism or satisfaction or efficiency or performance or guidelines* or behavi?r or quality))[tiab]

9. #6 NOT #5

10. #5 OR #7
11. #4 AND #8

B. Education II-(pre-service)

12. "pre service"   [tiab] or preservice [tiab] Or "Inservice Training"[Mesh] OR "continuing education"[tiab]

13. #4 AND #20

Human Resources for Health (All Categories)

14. #11 NOT #13

15. #13 NOT #11

16. #14 OR #15

Service Delivery

A. Access, integrated care, continuity of care, modes of delivery

1. "Delivery of Health Care"[Mesh:noexp] OR "Delivery of Health Care, Integrated"[Mesh:noexp] OR "Referral and Consultation"[Mesh:noexp] OR "After-Hours Care"[Mesh] OR "Health Care Reform"[Mesh:noexp] OR "Health Services Needs and Demand"[Mesh] OR ("Health Services Accessibility"[Mesh] AND ("Rural Health Services"[Mesh] OR "Adolescent Health Services"[Mesh] OR "Community Health Services"[Mesh] OR "Women's Health Services"[Mesh] OR "ambulatory care"[MeSH Terms:noexp] OR "Home Care Services"[Mesh] OR "Health Facilities"[Mesh] OR "Family Practice"[Mesh] OR "Nursing Services"[Mesh])) OR "Continuity of Patient Care"[Mesh] OR "telemedicine"[MeSH Terms] OR "telemetry"[MeSH Terms] OR ("Telephone"[Mesh] AND "Delivery of Health Care"[Mesh])

B. Role of non-sector sector

2. "Private practice" [MeSH] OR " physicians, family "[MeSH] OR " professional corporations "[MeSH] OR " organizations, nonprofit"[MeSH] OR "Outsourced services" [MeSH] OR "Social marketing" [MeSH]

3. "private sector"[tiab] OR "private practitioner"[tiab] OR Private practice[tiab] OR "private provider"[tiab] OR "private providers"[tiab] OR "private provision"[tiab] OR "Non state sector"[tiab] OR "Non state"[tiab] OR "non formal"[tiab] OR informal [tiab] OR traditional[tiab] OR licensed[tiab] OR "non licensed" [tiab]OR unlicensed [tiab] OR "drug vendors"[tiab] OR "medicine sellers"[tiab] OR pharmacists[tiab] OR "NGO"[tiab] OR "Public Private Partnership"[tiab] OR Franchising [tiab] OR Franchizing [tiab] OR Contract*[tiab] OR Contracts [tiab] OR Contracting [tiab]

4. #2 Not #3

C. <u>Quality of care and performance</u>

5. "health manpower" [MeSH]

6. “health personnel" [tiab] OR "health care personnel" [tiab] OR "healthcare personnel" [tiab] OR "medical personnel" [tiab] OR "health professional" [tiab] OR "health care professional*" [tiab] OR "healthcare professional*" [tiab] OR "medical professional*" [tiab] OR "health worker*" [tiab] OR "health care worker*" [tiab] OR "healthcare worker*" [tiab] OR "medical worker*" [tiab] OR "health workforce" [tiab] OR "health care workforce" [tiab] OR "healthcare workforce" [tiab] OR "medical workforce" [tiab] or “human resource” [tiab]

7. #6 NOT #5
8. #5 OR #7

9. "Quality Assurance, Health Care"[Mesh] OR "Quality Indicators, Health Care"[Mesh] OR "Quality of Health Care"[Mesh] OR "Health Care Quality, Access, and Evaluation"[Mesh] OR "Quality Control" [Mesh] OR "Total Quality Management" [Mesh] OR "Health Care Evaluation Mechanisms"[Mesh] OR “quality of care”[tiab] OR “clinical standards”[tiab] OR “structural quality”[tiab] OR “process quality”[tiab]

10. #8 AND #9

Service Delivery (All Categories)

11. (#1 NOT #4) AND (#1 NOT #10)

12. (#4 NOT #1) AND (#4 NOT #10)

13. (#10 NOT #1) AND (#10 NOT #4)

14. (#11) OR (#12) OR (#13)

Health Information systems

1. "Medical Records"[Mesh:NoExp] OR "Medical Records Systems, Computerized" [Mesh] OR "Medical Records, Problem-oriented" [Mesh] OR "Management Information Systems" [Mesh] OR "Drug Information Services" [Mesh]

Governance Arrangements

A. Government regulation and legislation

1. "Government Regulation"[Mesh] OR "Legislation, Medical" [Mesh] OR "Legislation, Drug" [Mesh] OR "Legislation, Hospital" [Mesh] OR "Legislation, Pharmacy" [Mesh] OR "Legislation, Nursing" [Mesh] OR "Facility Regulation and Control" [Mesh] OR

“committee*”[tiab] OR “accountab*”[tiab] OR “corrupt*”[tiab] OR “fraud” [tiab] OR “bribe” [tiab] OR “penalt*”[tiab] OR “gaming” [tiab] OR “game” [tiab] OR “coerc*”[tiab]

B. <u>Professional authority and roles (scope, content and location of practice)</u>

2. “Organizational Affiliation" [Mesh:NoExp] OR “Staff Development”[Mesh] OR "Professional Role"[Mesh] OR "Practice Guidelines as Topic"[Mesh] OR "Professional-Patient Relations" [Mesh] OR "Nurse-Patient Relations" [Mesh] OR "Physician-Patient Relations" [Mesh]

C. <u>Audit</u>

3. "Management Audit"[Mesh:NoExp] OR "Clinical Audit" [Mesh] OR “supervis*”[tiab] OR “verif*”[tiab]

D. <u>Consumer involvement</u>

4. "Consumer Participation"[Mesh]

Governance (All Categories)

5. (#1 NOT #2) AND (#1 NOT #3) AND (#1 NOT #4)
6. (#2 NOT #1) AND (#2 NOT #3) AND (#2 NOT #4)
7. (#3 NOT #1) AND (#3 NOT #2) AND (#3 NOT #4)
8. (#4 NOT #1) AND (#4 NOT #2) AND (#4 NOT #3)
9. #5 OR #6 OR #7 OR #8

Service utilisation, coverage, equity, and knock-on effects

1. “use-effectiveness”[mesh] OR “health services accessibility”[Mesh] OR “sustained use”[Mesh] OR “uptake”[Mesh] OR “up-take”[Mesh] OR “utilization”[Mesh] OR “service

utilisation”[Mesh] OR “patient acceptance of health care”[Mesh] OR “equity, health” [MeSH] OR “referral and consultation” [Mesh] OR “transportation” [Mesh]

2. “use-of-service”[tiab] OR “use of service”[tiab] OR “service use”[tiab] OR “effective use” [tiab] OR “sustained use”[tiab] OR “uptake”[tiab] OR “up-take”[tiab] OR “utilisation”[tiab] OR “service utilisation”[tiab] OR “health care utilisation”[tiab] OR “access”[tiab] or “transport*”[tiab] or “spillover”[tiab] OR “bypass*”[tiab] OR “uninten* effect”[tiab] OR “knock on effect”[tiab] OR “knock-on effect”[tiab] or “coverage”[tiab]
3. #1 OR #2

Low- and Middle-Income Countries

1. "Developing Countries"[Mesh] OR Africa[Mesh] or "Africa South of the Sahara"[Mesh] or Asia[Mesh] or "South America"[Mesh] or "Central America"[Mesh] OR Africa[tiab] or Asia[tiab] or "South America"[tiab] or "Latin America"[tiab] or "Central America"[tiab]

2. "American Samoa"[tiab] or Argentina[tiab] or Belize[tiab] or Botswana[tiab] or Brazil[tiab] or Bulgaria[tiab] or Chile[tiab] or Comoros[tiab] or Costa Rica[tiab] or Croatia[tiab] or Dominica[tiab] or Equatorial Guinea[tiab] or Gabon[tiab] or Grenada[tiab] or Hungary[tiab] or Kazakhstan[tiab] or Latvia[tiab] or Lebanon[tiab] or Libya[tiab] or Libia[tiab] or Libyan[tiab] or Lithuania[tiab] or Malaysia[tiab] or Mauritius[tiab] or Mexico[tiab] or Micronesia[tiab] or Montenegro[tiab] or Oman[tiab] or Palau[tiab] or Panama[tiab] or Poland[tiab] or Romania[tiab] or Russia[tiab] or Seychelles[tiab] or Slovakia[tiab] or South Africa[tiab] or "Saint Kitts and Nevis"[tiab] or "Saint Lucia"[tiab] or "Saint Vincent and the Grenadines"[tiab] or Turkey[tiab] or Uruguay[tiab] or Venezuela[tiab] or Yugoslavia[tiab] or Mayotte[tiab] or "Northern Mariana Islands"[tiab] or "Russian Federation"[tiab] or Samoa[tiab] or Serbia[tiab] or "Slovak Republic"[tiab] or "St Kitts and Nevis"[tiab] or "St Lucia"[tiab] or "St Vincent and the Grenadines"[tiab]

3. Albania[tiab] or Algeria[tiab] or Angola[tiab] or Armenia[tiab] or Azerbaijan[tiab] or Belarus[tiab] or Bhutan[tiab] or Bolivia[tiab] or "Bosnia and Herzegovina"[tiab] or Bosnia[tiab] or Cameroon[tiab] or China[tiab] or Colombia[tiab] or Congo[tiab] or Cuba[tiab] or Djibouti[tiab] or "Dominican Republic"[tiab] or Ecuador[tiab] or Egypt[tiab] or El Salvador[tiab] or Fiji[tiab] or "Georgia (Republic)" [tiab] or Guam[tiab] or Guatemala[tiab] or Guyana[tiab] or Honduras[tiab] or "Indian Ocean Islands"[tiab] or Indonesia[tiab] or Iran[tiab] or Iraq[tiab] or Jamaica[tiab] or Jordan[tiab] or Lesotho[tiab] or "Macedonia" [tiab] or "Marshall Islands"[tiab] or Micronesia[tiab] or "Middle East"[tiab] or Moldova[tiab] or Morocco[tiab] or Namibia[tiab] or Nicaragua[tiab] or Paraguay[tiab] or Peru[tiab] or

Philippines[tiab] or Samoa[tiab] or "Sri Lanka"[tiab] or Suriname[tiab] or Swaziland[tiab] or Syria[tiab] or Thailand[tiab] or Tonga[tiab] or Tunisia[tiab] or Turkmenistan[tiab] or Ukraine[tiab] or Vanuatu[tiab] or "Cape Verde"[tiab] or Gaza[tiab] or Georgia[tiab] or Kiribati[tiab] or Macedonia[tiab] or Maldives[tiab] or Palestine[tiab] or "Syrian Arab Republic"[tiab] or "West Bank"[tiab]

4. Afghanistan[tiab] or Bangladesh[tiab] or Benin[tiab] or "Burkina Faso"[tiab] or Burundi[tiab] or Cambodia[tiab] or "Central African Republic"[tiab] or Chad[tiab] or Comoros[tiab] or "Democratic Republic of the Congo"[tiab] or "Cote d'Ivoire"[tiab] or Eritrea[tiab] or Ethiopia[tiab] or Gambia[tiab] or Ghana[tiab] or Guinea[tiab] or Guinea-Bissau[tiab] or Haiti[tiab] or India[tiab] or Kenya[tiab] or Korea[tiab] or Kyrgyzstan[tiab] or Laos[tiab] or Liberia[tiab] or Madagascar[tiab] or Malawi[tiab] or Mali[tiab] or Mauritania[tiab] or Melanesia[tiab] or Mongolia[tiab] or Mozambique[tiab] or Myanmar[tiab] or Nepal[tiab] or Niger[tiab] or Nigeria[tiab] or Pakistan[tiab] or "Papua New Guinea"[tiab] or Rwanda[tiab] or Senegal[tiab] or "Sierra Leone"[tiab] or Somalia[tiab] or Sudan[tiab] or Tajikistan[tiab] or Tanzania[tiab] or East Timor[tiab] or Togo[tiab] or Uganda[tiab] or Uzbekistan[tiab] or Vietnam[tiab] or Yemen[tiab] or Zambia[tiab] or Zimbabwe[tiab] or Burma[tiab] or Congo[tiab] or Kyrgyz[tiab] or Lao[tiab] or "North Korea"[tiab] or "Solomon Islands"[tiab] or "Sao Tome"[tiab] or Timor[tiab] or "Vietnam"[tiab]

5. "developing country"[tiab] OR "developing countries"[tiab] OR "developing nation*"[tiab] OR "less* developed country"[tiab] OR "less* developed countries"[tiab] OR "under developed country"[tiab] OR "under developed countries"[tiab] OR "poor* country"[tiab] OR "poor* countries"[tiab]

6. "middle income country"[tiab] or "middle income countries"[tiab] or "low income country"[tiab] or "low income countries"[tiab]

7. lmic[tiab] or lmics[tiab]

8. (#1) OR (#2) OR (#3) OR (#4) OR (#5) OR (#6) OR (#7)

9. Japan[tiab] OR "United States"[tiab]

10. **(**#8) NOT (#9)

Study types

1. “Randomized controlled trial”[Mesh] OR “Time series analysis”[Mesh] OR “Controlled clinical trial”[Mesh] OR “controlled clinical trial” [Mesh] OR “cross-sectional studies”[Mesh] OR “case-control studies”[Mesh] OR “cohort studies”[Mesh] OR “pilot studies”[Mesh] OR “economics”[Mesh] OR “cost-benefit analysis”[Mesh] OR “cost control”[Mesh] OR “cost savings”[Mesh] OR “cost of illness”[Mesh] OR “review”[Mesh] OR “program evaluation”[Mesh] OR “pilot projects”[Mesh] OR “feasibility studies”[Mesh] or “qualitative research”[Mesh]
2. Random*[tiab] OR control*[tiab] OR evaluat*[tiab] OR effect* [tiab] OR experiment*[tiab] OR (time adj series)[tiab] (“pre test” or pretest or “post test” or posttest)[tiab] OR chang*[tiab] OR compar*[tiab] OR (random$ or control*)[tiab] OR (control adj3 (area or cohort? or compare? or condition or design or group? or intervention? or participant? or study))[tiab] OR ((evaluat* or prospective or retrospective) adj1 study)[tiab] OR ("quasi-experiment*" or quasiexperiment* or "quasi random*" or quasirandom* or "quasi control*" or quasicontrol* or ((quasi* or experimental) adj3 (method* or study or trial or design*)))[tiab] OR ("time series" adj2 interrupt*)[tiab] OR (intervention* or impact or effectiveness or efficacy or service* or outcome* or output or treatment* or management or program* or project*)[tiab] OR “cost utility”[tiab] OR (Cost* adj2 effective*)[tiab] OR cost-effective*[tiab] OR (cost adj3 utility)[tiab] OR cost-utilit*[tiab] OR “implementation”[tiab] or “implementation research”[tiab] OR “case study”[tiab]
3. #1 OR #2

Publication Dates and limiting to "Humans"

Publication Date: 01/01/1995 to 31/05/2017

1. Animals[mh]
2. Humans[mh]

3. #1 not (#1 and #2)
4. (#2) NOT (#3)
